# Genomic distribution of a novel *Pyrenophora tritici-repentis ToxA* insertion element

**DOI:** 10.1371/journal.pone.0206586

**Published:** 2018-10-31

**Authors:** Paula M. Moolhuijzen, Pao Theen See, Richard P. Oliver, Caroline S. Moffat

**Affiliations:** Centre for Crop Disease and Management, Department of Environment and Agriculture, Curtin University, Bentley, Western Australia, Australia; University of Nebraska-Lincoln, UNITED STATES

## Abstract

The *ToxA* effector is a major virulence gene of *Pyrenophora tritici-repentis* (Ptr), a necrotrophic fungus and the causal agent of tan spot disease of wheat. *ToxA* and co-located genes are believed to be the result of a recent horizontally transferred highly conserved 14kb region a major pathogenic event for Ptr. Since this event, monitoring isolates for pathogenic changes has become important to help understand the underlying mechanisms in play. Here we examined *ToxA* in 100 Ptr isolates from Australia, Europe, North and South America and the Middle East, and uncovered in isolates from Denmark, Germany and New Zealand a new variation, a novel 166 bp insertion element (PtrHp1) which can form a perfectly matched 59 bp inverted repeat hairpin structure located downstream of the *ToxA* coding sequence in the 3’ UTR exon. A wider examination revealed PtrHp1 elements to be distributed throughout the genome. Analysis of genomes from Australia and North America had 50–112 perfect copies that often overlap other genes. The hairpin element appears to be unique to Ptr and the lack of ancient origins in other species suggests that PtrHp1 emerged after Ptr speciation. Furthermore, the *ToxA* UTR insertion site is identical for different isolates, which suggests a single insertion event occurred after the *ToxA* horizontal transfer. *In vitro* and *in planta*-detached leaf assays found that the PtrHp1 element insertion had no effect on *ToxA* expression. However, variation in the expression of *ToxA* was detected between the Ptr isolates from different demographic locations, which appears to be unrelated to the presence of the element. We envision that this discovery may contribute towards future understanding of the possible role of hairpin elements in Ptr.

## Introduction

The necrotrophic fungus *Pyrenophora tritici-repentis* (Ptr) is the causal agent of tan spot of wheat, a major disease that causes significant losses to the wheat industry worldwide [1]. The pathogen produces at least three effectors (host-selective toxins), namely ToxA, ToxB and ToxC, which induce necrosis or chlorosis in host genotypes harbouring the corresponding sensitivity gene [2, 3]. ToxA is the predominant Ptr effector, prevalent in the majority of isolates worldwide [4–7]. Upon exposure to ToxA, wheat varieties that possess the ToxA sensitivity gene *Tsn1* exhibit necrosis, leading to a reduction in photosynthesis ultimately impacting grain production [8]. *ToxA*/*Tsn1* is strongly associated with tan spot disease [9]. Highly similar *ToxA* gene are also found in some isolates of *Parastagonospora nodorum* [10] and *Bipolaris sorokiniania* (Bs) [11] as the result of horizontal transfer events. *P*. *nodorum ToxA* has 15 different haplotypes (H1-H15) with single nucleotide polymorphism (SNP) variations at 25 nucleotide sites; of these 18 sites have non-synonymous changes [12, 13]. In *PtrToxA*, only three SNP based haplotypes (H14-H16) have been reported and two SNP sites give rise to non-synonymous amino acid changes. H15 is the only *PtrToxA* haplotype observed in Australia [14], while haplotypes H14 and H16 have only been reported in Europe [13].

As *ToxA* is a major virulence gene in tan spot, it is important to monitor potential gene changes, which may indicate a shift towards a more potent haplotype, as well provide relevant sequence resources for variants. Hence we have screened isolates of Ptr for any changes in the *ToxA* gene region. In this study we report the discovery of a variant *ToxA* hairpin element that is unique but not conserved in the Ptr genome, and examined the impact of this element on *ToxA* expression.

## Results

### PCR analysis of *ToxA* in European and New Zealand *P*. *tritici-repentis* isolates

**Fig 1:**
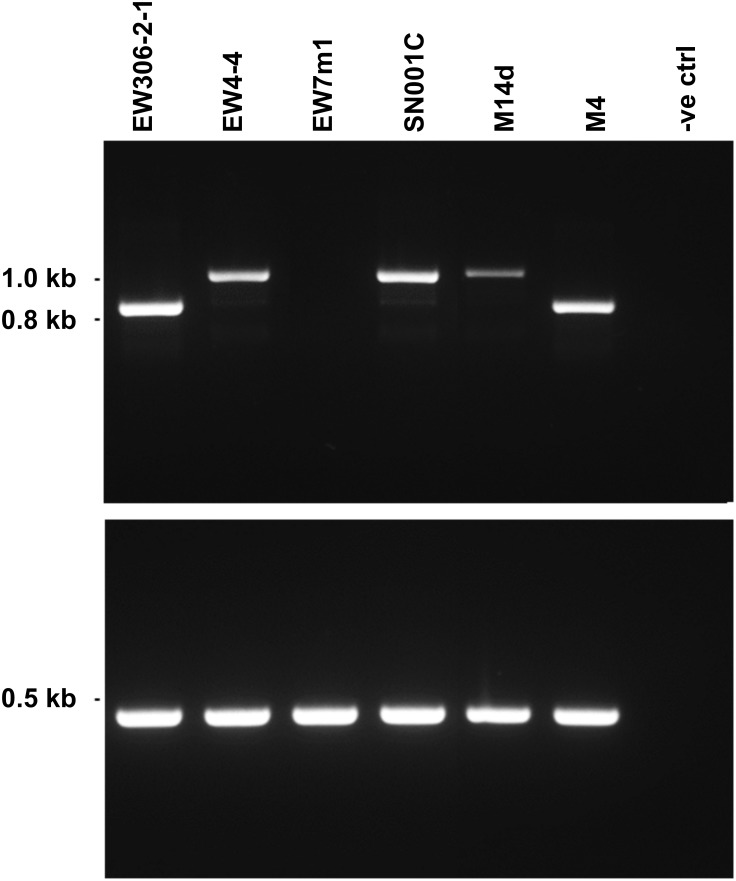
Gel electrophoresis showing variation of the *ToxA* gene amplicon sizes between Ptr isolates from various demographic locations. Top gel shows, a larger than expected product size of approximately at 1 kb amplified in three isolates (EW4-4, SN001C and M14d). Isolates EW306-2-1 and M4 amplified the expected product sized of 832 bp, while *ToxA* was not detected in EW7m1. No template was used as a negative control. Bottom gel shows DNA amplification of a 490 bp region that is unique in Ptr genome [16] that was included as a positive control.

Ptr isolates from New Zealand (M14d), Denmark (EW13061 (EW306-2-1), EW4-4, and EW7m1) and Germany (SN001C) were examined for variation in the *ToxA* locus using PCR primers that amplified the coding region and 256 bp down stream. The Australian isolate M4 was included as a control [14, 15]. A PCR amplification product of approximately 1 kb size was detected in EW4-4, M14d and SN001C, as compared to the expected 842 bp product size found in EW306-2-1 and M4 (Fig 1). No amplification was detected for isolate EW7m1, indicating absence of the *ToxA* gene, therefore EW7m1 is not a race that carries the 14 kb horizontally transferred *ToxA* region. A faint band with a similar product size to the expected *ToxA* amplification was also observed for isolates that amplified the larger PCR product, an artefact on the PCR amplification around the hairpin. To our knowledge, this variation in *ToxA* PCR product size has not been previously identified in any isolates to date.

### Cloning of the *ToxA* gene region

**Fig 2:**
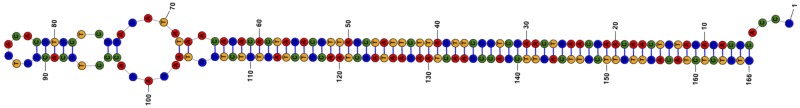
The *ToxA* insertion element PtrHp1 (166 bp) has a secondary hairpin structure.

To investigate the discrepancy between the sizes of *ToxA* gene PCR products, a high-fidelity PCR amplification was performed on M14d DNA using primer ToxA1630F and ToxA1630R that targeted a 1.6 kb region of the *ToxA* locus. In agreement with the gel analysis (Fig 1), cloning of the *ToxA* PCR product produced two clones with different insert sizes (S1 Fig). Sanger sequencing of the larger insert produced very short DNA sequence that displayed a sharp drop in sequencing signal (S1 Fig). Subsequently, the two plasmids were sequenced with an Illumina Miseq and assembled into single contigs M14d_2 (clone1) and M14d_3 (clone2) of lengths 1.83 kb and 1.63 kb, respectively. Sequence alignment between the M14d_2 clone insert and M4 *ToxA* locus identified a 166 bp insertion that contained a palindromic sequence of 59 bp (S1 Fig). This insertion, when analysed for secondary structure displayed a hairpin element, designated as PtrHp1 (Fig 2). M14d_3 did not have the PtrHp1 insertion and appeared to be a PCR artefact caused by the hairpin.

### Ptr *ToxA* region sequence analysis

To further investigate the hairpin element in the Ptr genomes, four isolates with the variant *ToxA* gene region (EW306-2-1, EW4-4, EW7m1 and SN001C) were genome sequenced with Illumina Hi-seq technology and assembled. The *ToxA* regions were then extracted and aligned to Ptr *ToxA* isolate M4 to identify the sites of variations [14] (S2 Fig). The annotated *ToxA*-region sequences from all isolates have been submitted to NCBI GenBank and can be found under Accession numbers MH017415, MH017417 and MH017418 (Table 1).

**Table 1:** Isolate source and *ToxA* assembly.

Isolate	NCBI Gene Accession	Country of origin	Year collected	*ToxA* gene length (bp)
**EW306-2-1**	MH017415	Denmark	2015	1,119
**EW4-4**	MH017417	Denmark	2015	1,285
**EW7m1**	-	Denmark	2015	Absent
**SN001C**	MH017418	Germany	2016	1,285

**Fig 3:**
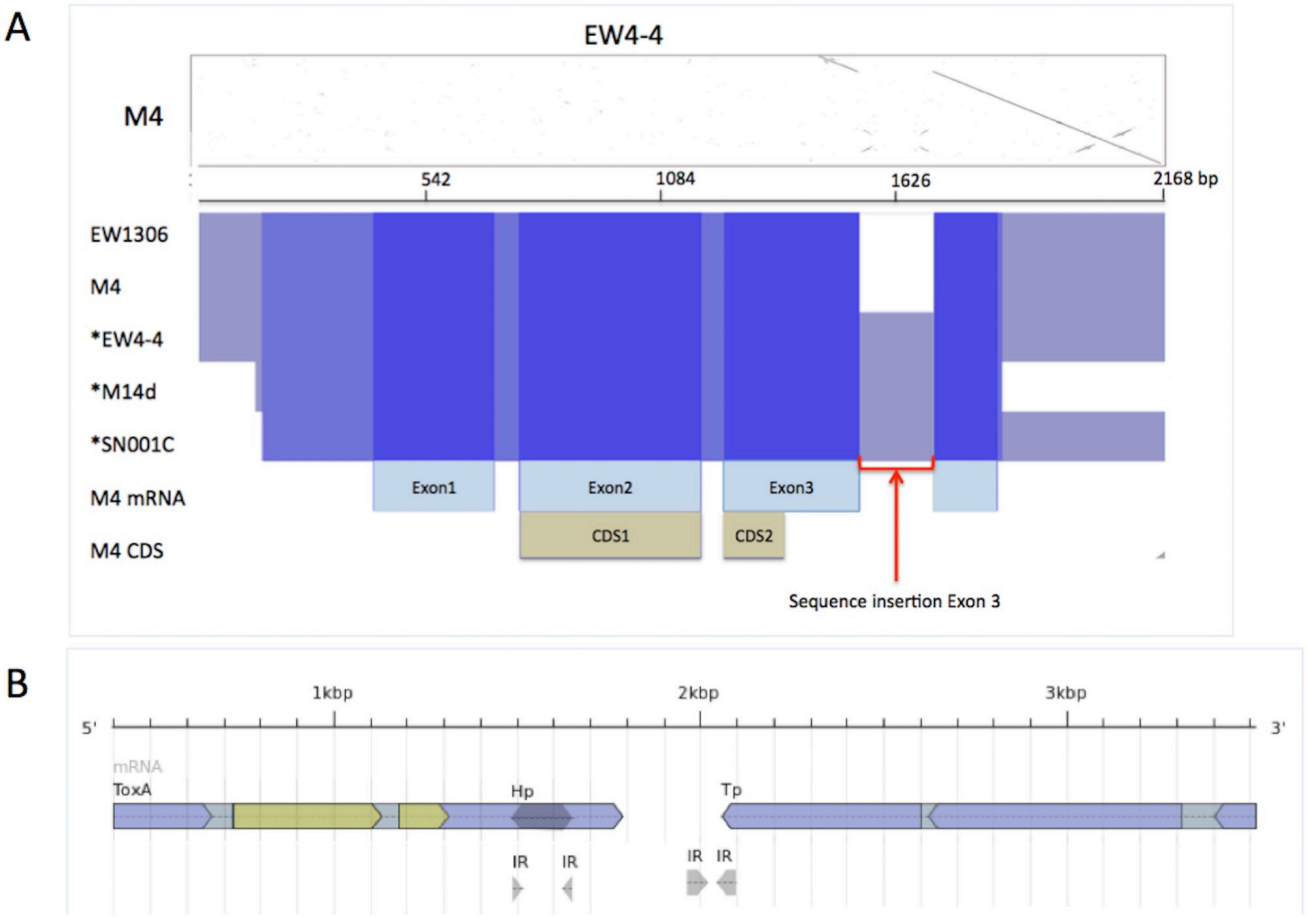
*P*. *tritici-repentis ToxA* region nucleotide alignment. (A) The sequence plot of EW4-4 (horizontal axis) and M4 (vertical axis) shows a deletion site in M4 (Contig1 5,732,328–5,732,329 bp) and an insertion (166bp) in EW4-4 between 1,484 and 1,649 bp inclusively. An EW4-4 inverted repeat downstream of the insertion site is visible at sequence positions 1,930 to 2,094bp. Under the dot plot an overview of the *ToxA* region (nucleotide multiple sequence alignment) shows the 3’UTR 166 bp insertion for isolates EW4-4, M14d and SN001C. Sequence alignment homology is shown (blue) and deletion (dotted line). *Asterisk indicates *ToxA* variants. (B) Ptr M4 *ToxA* and *Parastagonospora nodorum Tp* transposase (orthologue of SNOG16572) genes aligned to EW4-4 nucleotide region (4 kb) show the downstream inverse repeat (IR) position. *ToxA* coding sequence is CDS is shown in green and mRNA in blue.

The *ToxA* regions of isolates EW306-2-1, EW4-4, M14d, M4, SN001C, M4 mRNA (transcript) and M4 CDS were aligned to M4 (haplotype H15) [14]. In line with the findings from the PCR analysis, an identical insertion (166 bp) was identified in the 3’ UTR of *ToxA* exon3 for isolates EW4-4, M14d and SN001C, and the absence of sequence in the region is shown as a dotted line (Fig 3A). Intriguingly, the sequence plot between the PtrHp1 *ToxA* variant and M4 also revealed the insertion contained short terminal inverse repeats (TIRs) with approximately 40 bp sequence similarity to an inverse repeat (IR) element (Fig 3A). The variant sequence self- plot and variant versus M4 sequence plot can be found in S2 Fig.

The *ToxA* downstream IR element is found in the intergenic region between *ToxA* and a transposase companion gene (*Tp*) (an orthologue of *P*. *nodorum* SNOG16572) (Fig 3B). The positions of *ToxA*, IR and Tp for EW4-4 are shown in Fig 3B. The PtrHp1 and the IR shared only short terminal repeats of 20 bp.

**Fig 4:**
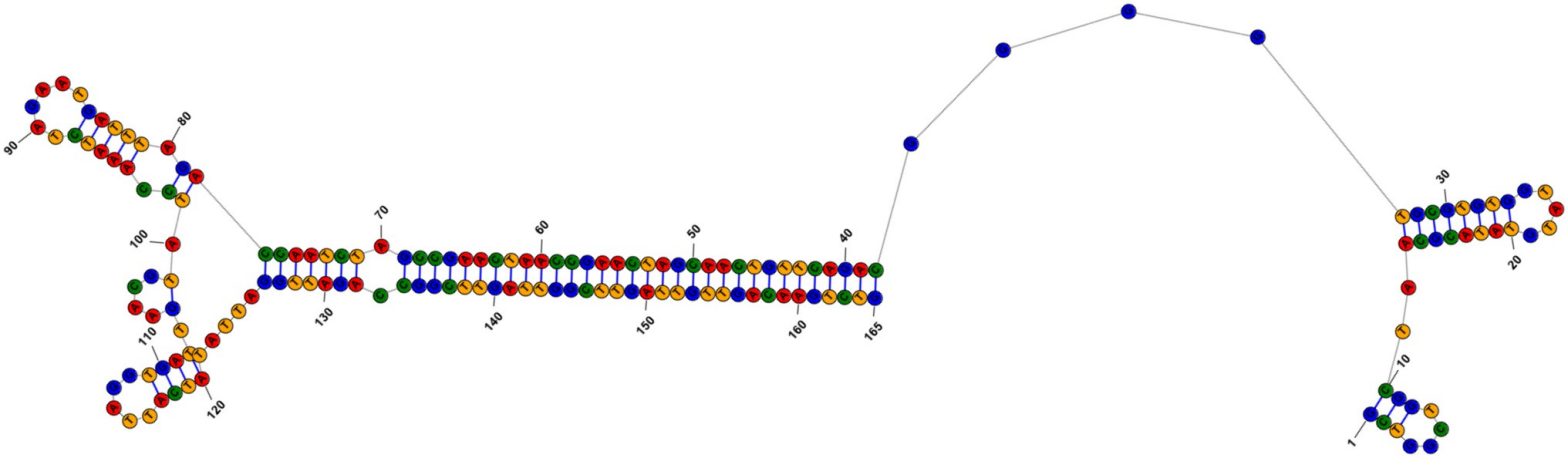
Predicted secondary structure of the intergenic inverse repeat (IR). The figure shows the predicted secondary structure of the intergenic inverse repeat found downstream of the *ToxA* PtrHp1 element insertion site.

To further explore any structural similarity between PtrHp1 and the intergenic IR, a 165 bp region containing the IR was extracted from the EW4-4 genome sequence (EW4-4: 1,490–1,647 bp) and analysed for secondary structure prediction. PtrHp1 and the IR did not share structural similarity (Fig 4).

### Distribution of transposase associated with *ToxA*

Transposases disperse throughout the genome and are often associated with effectors, as seen for *ToxA*. We therefore investigated the presence of the *ToxA* companion element *Tp* in Ptr and a number of closely related Pleosporales species to determine if any other pathogenic or effector-like factors were associated with it and if there was a correlation between the sites of the elements.

#### Genomic distribution of Ptr *ToxA Tp*

The *ToxA* horizontally transferred genomic region, highly conserved within other *ToxA* containing species Bs and Pa, also contains a conserved *Tp* (449 amino acid protein) characterised by two Ribonuclease H-like domains SSF53098 (IPR012337). The *Tp* sequence was searched in the available Pleosporales genomes. All *ToxA* containing isolates of Ptr, Bs and *P*. *nodorum* possessed only a single high identity (> = 99%) *Tp* associated with the *ToxA* as expected (S1 Table). However, *Pyrenophora* species (Ptr and *P*. *teres f*. *teres* (Ptt)) also possessed extra *Tp-like* copies with lower sequence similarity. It is worth noting that non-*ToxA* Ptt and Ptr (SD20) possessed only a single lower identity *Tp-like* copy. The lower identity *Tp-like* copies in Ptr and Ptt appear to be ancestral *ToxA*-unrelated genes.

#### Genomic distribution of *ToxA* PtrHp1

The distribution of the PtrHp1 element was then explored in the genome of M4 and another 39 available Pleosporales genomes. The PtrHp1 element was found to be specific to Ptr (S1 Table), and 105 identical copies were identified throughout the M4 genome. All Ptr isolates were found to have between 50–112 perfect copies except Ptr isolates SD20 and DW7, a probable artefact of the shorter 75 bp pair end read assemblies. PtrHp1 copies were also found associated with another 41 genes, of which 21 were hypothetical proteins. Gene ontology analysis identified molecular functions associated with protein dimerization, dipeptidyl-peptidase and hydrolase activities, and DNA and protein binding. Biological processes identified were associated with cleavage involved in rRNA processing, DNA integration and microtubule-based processes. None of the genes had predicted effector or secretion properties according to EffectorP and SignalP respectively [17, 18] (S2 Table). Although no PtrHp1 elements were found (at greater than 90% sequence identity) in the other Pleosporales genomes, short TIRs of up to 40 bp of PtrHp1 were identified (S4 Fig). The distribution of the PtrHp1 element compared to the distribution of the low identity *Tp* copies showed no specific correlation between the two elements (S4 Fig).

#### Geographic distribution of PtrHp1 *ToxA* insertion

To determine the possible geographic distribution of the *ToxA* PtrHp1 insertion, a global collection of 100 Ptr DNA samples from *ToxA* containing isolates were PCR tested. A total of 26 *ToxA* isolates contained a PtrHp1 insertion. The detected insertions were only found in isolates from 3 countries (Denmark, Germany, New Zealand) of the 12 countries tested (S3 Fig).

#### Evaluation of *ToxA* expression in PtrHp1 isolates *in vitro* analysis

**Fig 5:**
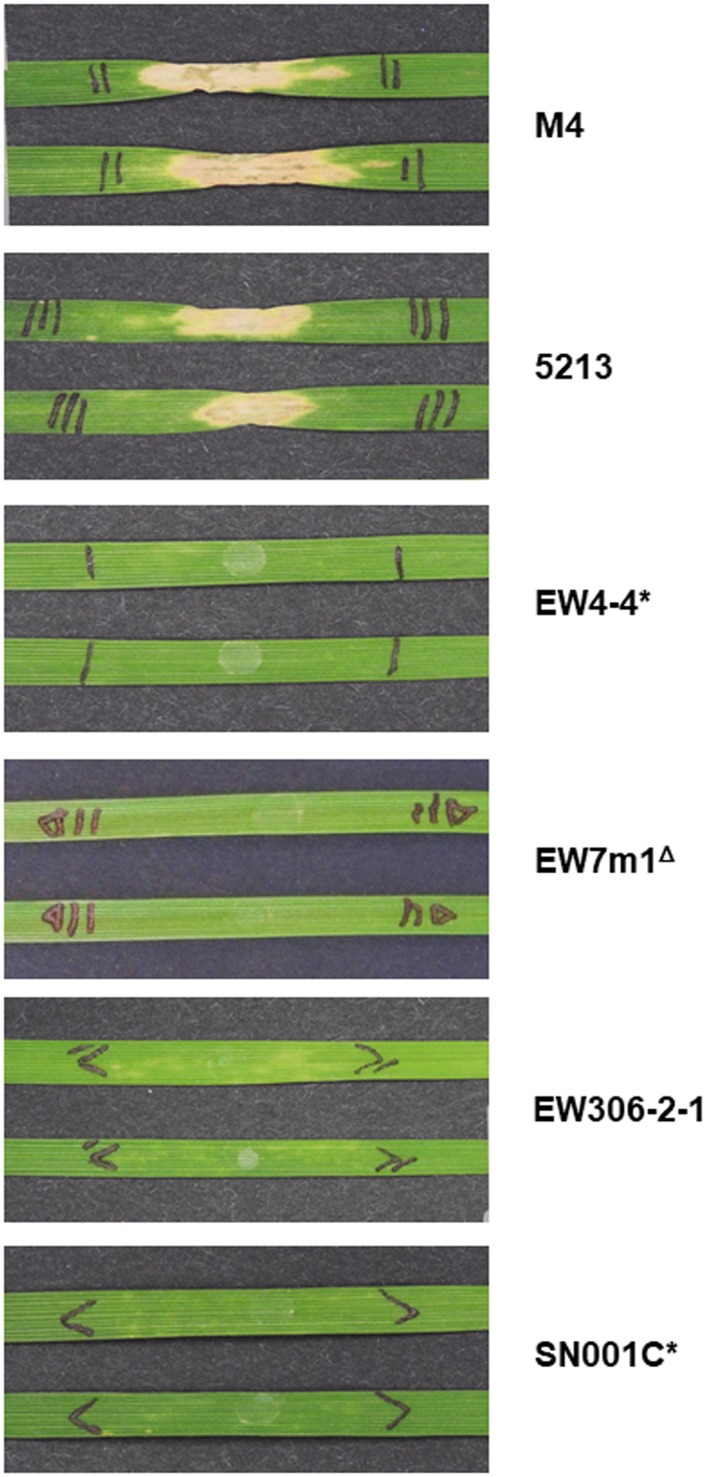
Culture filtrate activity of Ptr isolates with or without the PtrHP1 element on the ToxA sensitive wheat variety Yitpi. * Isolates with the PtrHp1 element; Δ Isolates without the *ToxA* locus. Photographs were taken 10 days post-infiltration.

To determine if the 3’ UTR PtrHp1 element had any effect on *ToxA* expression, Ptr isolates containing the PtrHp1 element were cultured *in vitro* in Fries 3 media, standard conditions for the production of ToxA. A plant bioassay performed using the culture filtrate from isolates showed that only the Australian isolates produced enough ToxA to induce necrosis on the ToxA-sensitive wheat cultivars (Fig 5). No necrotic symptom was observed on the *Tsn1* sensitive cultivar for EW306-2-1, EW7m1 and the PtrHp1-containing isolates EW4-4 and SN001C.

Quantitative gene expression analysis (Table 2) and the absence of ToxA protein bands on the SDS-PAGE analysis of secreted proteins for isolates EW4-4, EW306-2-1 and SN001C (S5 Fig) indicated that the absence of observable necrotic symptoms was due to the low expression of *ToxA* gene in the *in vitro* culture of European Ptr isolates regardless of *ToxA* PtrHp1 absence or presence.

**Table 2:** Ptr *ToxA* gene expression levels for *in vitro* culture. Gene expression levels were normalized to *Actin* gene expression.

Isolate	Normalized gene expression
	*ToxA*
M4	134.89 ± 35.04^a^
5213	40.09 ± 7.25^b^
EW306-2-1	0.11 ± 0.02^b^
EW4-4*	0.05 ± 0.01^b^
SN001C*	0.11 ± 0.02^b^

^ab^ same lettering are not significant (Tukey Kramer HSD test, p < 0.05). Data represent mean ± SE for five biological replicates.

* Asterisk indicates isolates with the PtrHp1 element.

#### Evaluation of *ToxA* gene expression in PtrHp1 isolates by *in planta* analysis

**Fig 6:**
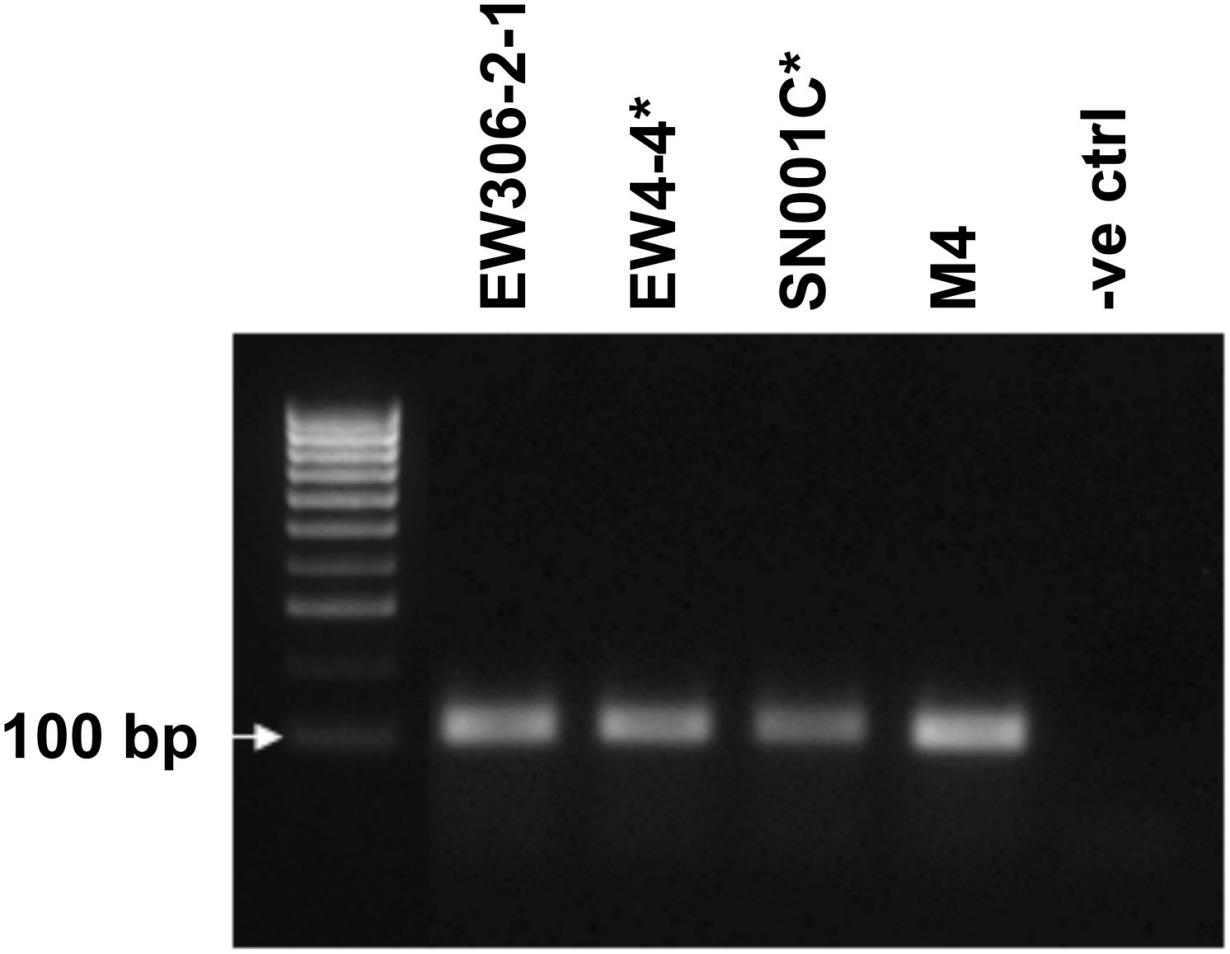
*In planta* expression of *ToxA* during infection on Yitpi *Tsn1* detached leaves assay.

The *ToxA* gene expression was further investigated *in planta* via detached leaf assays (spores inoculated on detached leaf) to determine if the PtrHp1 element had any effect on the regulation of *ToxA* during host infection. The *ToxA* transcript was detected 3 days post-inoculation for isolates both with and without the hairpin element (Fig 6) indicating that the insertion of the hairpin element at the 3’UTR did not disrupt the expression of *ToxA*.

Quantitative gene expression analysis showed that *ToxA* expression was variable between isolates, with the European Ptr isolates EW4-4 and SN001C (which both contain PtrHP1) and EW306-2-1 displaying a lower level of *ToxA* expression in comparison to M4 (Table 3).

**Table 3:** *In planta* gene expression of *ToxA* in Ptr isolates on *Tsn1* Yitpi wheat variety. Gene expression levels were normalized to *Actin* gene expression.

Isolate	Normalized gene expression
	*ToxA*
M4	349.13 ± 19.64^a^
EW306-2-1	64.21 ± 52.89^b^
EW4-4*	24.90 ± 8.09^b^
SN001C*	53.59 ± 26.78^b^

^ab^ same lettering are not significant (Tukey Krammer HSD test, p < 0.05). Data represent mean ± SE for four biological replicates.

* Asterisk indicates isolates with the PtrHp1 element.

## Discussion

Fungal pathogen genomes have been shown to be highly plastic in nature [14, 19, 20] and variant changes occur frequently, as seen in fungicide resistance and resistance gene breakdown [21]. The characterisation of gene structure and neighbouring genomic elements is important to monitor and understand variant changes and possible effects on gene regulation. The structure of the *ToxA* region in Pa, Ptr and Bs isolates has remarkably little variation, consistent with the idea that the horizontal gene transfer events were very recent [12, 14]. In this study a large hairpin element insertion into the Ptr *ToxA* gene 3’ UTR was identified for the first time.

It has been reported that the UTRs of many genes can have regulatory regions that post-transcriptionally influence gene expression [22]. It is therefore possible that motifs, mainly RNA secondary structures, in the 3′ UTRs regulatory region bind specific proteins to bring about gene regulation [22, 23]. It is also possible that the insertion of a well-defined hairpin element such as PtrHp1 could be a mechanism for gene silencing. For example, dsRNA/hairpin RNA generate 21–25 nt mature siRNA or miRNA duplexes, that result in silencing either by mRNA cleavage or translational repression [24]. Given that a PtrHp1 insertion was detected in the 3’UTR of *ToxA*, it was necessary to determine if PtrHp1 would alter *ToxA* gene expression. *ToxA* expression was notably lower for all the European isolates compared to the Australian isolates by *in vitro* assays. Despite the lower expression of *ToxA* it appeared that the hairpin insertion variation did not impact the regulation of *ToxA*. However variation in the expression levels observed between the different isolates warrant further investigation. It is yet to be determined if PtrHp1 belongs to another Ptr specific gene regulatory system.

This is also the first report of *Tp* low identity copies in *ToxA* and non-*ToxA* Ptr and Ptt isolates. The single high identity *Tp* found in *P*. *nodorum* and Bs isolates is likely from a recent horizontal transfer. It does however appear that *Pyrenophora* species uniquely have genomic evidence of possible ancestral invasion events or an ancestral family related to the *Tp*. In contrast, PtrHp1 element appears to be dispersed throughout Ptr genomes resulting in many perfect copies. Full length elements are absent in other species and only short TIRs sequences were identified, which for the most part shared sequence identity to different inverse repeat families. It is possible that small sequence signatures identified in *B*. *cookei*, *B*. *ma*, *L*. *maydis* and *P*. *seminiperda* are evidence of ancient element origins (S4 Fig), otherwise the lack of full PtrHp1-like elements in closely related species suggests PtrHp1 has appeared after divergence of the species, estimated 8 million years ago [25].

In the case of the hairpin insertion event into *ToxA* 3’UTR, different isolates share an identical PtrHp1 insertion site, which suggests that a single insertion event has occurred after the horizontal transfer of the *ToxA* to Ptr first reported in 1941 [10].

Although *ToxA* has been detected in isolates around the world, studies typically tested only a few isolates at a time. Ptr population studies in almost all cases did not perform *ToxA* PCRs, with the exception of a few larger studies that did not amplify PCR products that would capture the 3’UTR PtrHp1 insertion site [26–28]. Our primer sets present a new resource for the *ToxA* downstream region. The global distribution of this element has therefore yet to be determined and requires broader sampling with larger numbers of isolates to determine prevalence of PtrHp1 in *ToxA*. It is however possible that the geographic occurrence of the hairpin element in only North West Europe and New Zealand could be a result of shuttle breeding, a practice of growing cultivars in two contrasting climatic growing conditions [29].

## Conclusions

Monitoring pathogenic gene changes is important to ascertain if evolutionary pressures are in play. A substantial variant to the Ptr *ToxA* gene has been identified as a Ptr specific hairpin element, PtrHp1, inserted into the 3’ UTR. The new element however did not appear to affect *ToxA* expression. We envision that this discovery may contribute towards future understanding of the possible role of hairpin elements in Ptr.

## Materials and methods

### Isolate collection and DNA extraction

Ptr isolates EW306-2-1, EW4-4, and EW7m1 were collected from Denmark SN001C from Germany, and M4 and 5213 from Australia. Fungi were routinely grown on V8PDA agar according to Moffat et al., 2014. Genomic DNA was extracted according to Moolhuijzen, et al. 2018. To detect the geographic distribution of PtrHp1, a collection of Ptr genomic DNA containing the *ToxA* gene was used which consists of DNA from Australia (5 isolates, 1987–2009), Brazil (24 isolates, 2007), Canada (2 isolates, 1980s), Czech Republic (1 isolate, 2000), Denmark (11 isolates, 2006–2015), Germany (9 isolates, 2014–2016), Hungary (1 isolate, 2006), Iran (15 isolates, 2010–2011), Mexico (8 isolates, 1992–2008), New Zealand (12 isolates, 2013–2014), Uruguay (1 isolate, 1998) and USA (16 isolates, 1978–2000). The Faculty of Agriculture and Life Sciences, Lincoln University New Zealand kindly provided Ptr genomic DNA of M14d.

### PCR and gel electrophoresis

The *ToxA* gene was amplified from genomic DNA using primer pair ToxAscreeningF 5’ CCTCGTACTTCTTTTCAGCG 3’ and ToxAscreeningR1 5’ TGTAGAAGACAAGATTTTGA 3’. PCR products were visualized by gel electrophoresis on 1.5% agarose gel and stained using SYBR safe DNA Gel stain (Life Technologies, Carlsbad, CA, USA). The 1630 bp region containing the ORF of the *ToxA* gene was amplified with ToxA1630F 5’ ACCATAGGCGACCGAGTAGA 3’ and ToxA1630R 5’ GATGGCGCCCGTGATAAATG 3’ using iProof High-Fidelity Master Mix (Bio-Rad, Hercules, CA, USA) as described in Moffat et al., 2014 [15]. The PCR product was gel-extracted and cloned into pGEM-T Easy (Promega). Plasmids were recovered from *E*. *coli* cultures using the GenEluteTM HP plasmid Miniprep Kit (Sigma) and digested with *EcoR*I (NEB) to confirm the PCR insert size. Sequencing reactions were performed as described in Moffat et al., 2015 [30]. The Australian Genome Research Facility (AGRF) sequenced the entire plasmids via Illumina Mi-Seq.

### Isolate sequencing

The Australian Genome Research Facility (AGRF) sequenced genomic DNA via Illumina Hi-Seq. Illumina sequence data for isolates was quality checked with FASTQC [31], trimmed for poor quality, ambiguous bases, and adapters using Skewer [32] and Trimmomatic v0.22 [33] with head crop 6 bp and minimum length 50 bp. *De novo* assembly was completed with SPAdes version v3.10.0 [34].

### ToxA sequence analysis

A genomic region containing *ToxA* (2 kb) was extracted from the *Pyrenophora tririci-repentis* genome M4 (Moolhuijzen, See et al. 2018), Contg1: 5730848–5732849 using EMBOSS Extractseq version 6.6.0.0 [35]. To retrieve the corresponding regions in the remaining genomes, the M4 2 kb region was then aligned to the other genomes using BLAT [36] with the option fastMap and the corresponding regions extracted with alignment greater 1500 bp.

The 2 kb nucleotide sequences and the M4 *ToxA* mRNA and CDS nucleotide sequences (NCBI Accession NQIK00000000) were then aligned using MUSCLE v3.8.31 [37, 38]. The 2 kb multiple sequence alignment was then visualised in JALView version 2.8.2 [39].

A 4 kb region was extracted from EW4-4, M4 *ToxA* and the *P*. *nodorum* isolate SN15 SNOG_16572 (NCBI Accession XM_001806616.1) transposase protein sequence (associated with *ToxA*) were then aligned using exonerate protein to genome [40]. The alignments were then viewed with GenomeTools Sketch version 1.5.1 [41, 42].

The *P*. *nodorum Tp* (SNOG16572) domain searches were conducted with InterProScan version 5.17–56 [43].

### Nucleotide secondary structure

The *ToxA* insertion element hairpin secondary structure was predicted using RNAstructure version 6.0 and drawn with StructureEditor 6.0 [44] predicting a maximum free energy (MFE) structure, with maximum expected accuracy, and pseudoknot prediction. Options selected—DNA—loop 30—maximum 20—percent 10—temperature 310.15—window 3.

### Genome analysis

*Pyrenophora tritici-repentis* genome sequence data was sourced from isolate M4 (NCBI Accession: NQIK00000000), BFP (NCBI Accession: AAXI00000000.1) [45]. Other Pleosporales (*Taxonomy ID*: 92860) genomes were downloaded from NCBI GenBank Genome division for comparative analysis, *Bipolaris maydis* ATCC 48331 (https://www.ncbi.nlm.nih.gov/genome/2586), *Bipolaris zeicola* (https://www.ncbi.nlm.nih.gov/genome/13436), *Pyrenophora seminiperda* (https://www.ncbi.nlm.nih.gov/genome/16916), *Pyrenophora teres f*. *teres* 0–1 (https://www.ncbi.nlm.nih.gov/genome/2995) [46]. The *Parastagonospora nodorum*, SN15 [47], LDSN03-Sn4, Sn79-1087 and Sn2000 genomes [48] were sourced from NCBI GenBank. The assembled genome of *Bipolaris sorokiniana* [11] was sourced from a previous study [14].

### Genome sequence alignments

The *P*. *nodorum* isolate SN15 SNOG_16572 (NCBI Accession XM_001806616.1) transposase gene protein sequence (associated with *ToxA*) was searched against unmasked genome data sets using BLATX [36] and minimum protein identity of 30%. Alignments were then filtered at 70% protein identity for higher identity reporting. The nucleotide insertion hairpin element genome searches were conducted with BLAT at greater than 90% sequence identity. The M4 genomic positions of the PtrHp1, Tp and IR-IE were mapped with Emboss lindna version 6.6.0.0.

The M4 gene annotations and the PtrHp mapped sites in M4 were examined for overlap using BedTools intersect version 2.17.0 [49, 50]. M4 gene protein Pfam [51] domain annotation at an expected value (e-value) ≤ 1e-05 was linked to gene ontologies using pfam2go [52, 53].

### Production of ToxA in vitro and plant bioassays

*In vitro* fungal cultures and plant bioassays were prepared as described [15]. One-week-old fungal cultures were filtered through gauze, sterilised using 0.22 μm membrane filter unit (Millex, Germany) and dialysed in 20 mM sodium phosphate buffer pH8.0. The culture filtrate was then used to infiltrate the first leaves of the *ToxA* sensitive Australian wheat cultivar Yitpi. Infiltrated leaves were evaluated for ToxA sensitivity 10 days post-infiltration. Fungal mycelia samples were harvested from the culture, immediately snap-frozen in liquid nitrogen and stored at -80°C for RNA extraction.

To analyse the secreted proteins of the culture filtrate, proteins were extracted from three-week old Fries 3 culture filtrate in trichloroacetic acid /acetone solution (6% (v/v) TCA) followed by solubilisation of protein in 50 mM Tris pH 8.0. The concentration of protein was estimated using bicinchoninic acid (BCA) protein assay [54]. Ten microgram of purified protein was analysed on SDS-PAGE as described [15].

### ToxA gene expression analysis

*In planta* gene expression analysis was performed on a detached leaf assay (cv. Yitpi). The second leaves of 2-week-old seedlings were excised and the ends of the leaves were submerged into water agar (15% (v/w) agar) containing 70 mg/L benzimidazole. Methods for producing spore inoculum were carried out as described [15]. Individual leaves were inoculated with 10 μl microliter of spore suspension (1500 spores / mL) and incubated under 12-h photoperiod at 22°C. Leaves were collected 3-days post-inoculation and immediately snap-frozen for RNA extraction.

Total RNA extraction for fungal mycelia and inoculated leaves were performed [15]. Quantitative *ToxA* gene expression was performed as described in [55] using primer pair ToxAFc TAAACGCCGATACAGTGCGA and ToxARa AAAGCTCATAAACGTCCCCC to amplify the *ToxA* gene. For the housekeeping actin gene (Act1), primer pair Act1F2 AGACCTTCAACGCTCCCGCC and Act1R2TGGCGTGGGGAAGAGCGAAAC was used. Gene expression data for the *in vitro* Fries culture and detached leaf assay were obtained from five biological replicates and four biological replicates, respectively. All statistical analyses were performed using JMP v 11.0.0 software. ANOVA was used to compare the means of the relative gene expressions of *ToxA*.

## Supporting information

S1 Fig Clonal product support. (A) Clone product amplification for M14d, (B) Sanger read base signals for clone sequence of M14d, (C) Sequence alignment of M4 *ToxA* gene region and M14d clone 1 and clone 2, the clone 1 palindromic sequence is shown as a grey bar.
(PDF)

S2 Fig *ToxA* region comparative analysis. A) *Pyrenophora tritici-repentis* (Ptr) *ToxA* region nucleotide multiple sequence alignment shows the 166bp sequence insertion in isolates EW4-4 and SN001C. B) *Pyrenophora tritici-repentis* (Ptr) *ToxA* region (~2kb) nucleotide sequence plot shows the 166bp sequence insertion in isolates CC142 *ToxA* 3’ UTR and downstream intergenic inverse repeat element (IR). a) CC142 self plot and b) CC142 on the horizontal axis and M4 on the vertical axis. CC142 *ToxA* mRNA UTRs (red) and CDS (green) are displayed on the bottom axis.
(PDF)

S3 Fig The detection of PtrHp1 element in *ToxA* Ptr isolates from various geographical locations. Countries with PtrHp1 detected in isolates are shown in red and in grey if not detected.
(PDF)

S4 Fig Pleosporales sequence similarity to PtrHp1 and PtrHp genomic distribution. A) Pleosporales sequence similarity to PtrHp1. Ptr race 4 (SD20), *B*.*zeicola* (Bze), *P*. *teres teres* (Ptt), *P*. *nodorum* (Sn15, Sn2000, Sn4, Sn79), *B*. *cookie* (Bco), *Z*. *tritici* (Ztr), *B*. *sorokiniana* (Bso), *L*. *maculans* (Lma), *P*. *seminiperda* (Pse) and *B*. *maydis* (Bma). B) The distribution of Ptr elements PtrTp (Tp blue), the IR-IE (IR red) and PtrHp (Hp black) are shown in M4 genome.
(PDF)

S5 Fig Sodium dodecylsulphate-polyacrylamide gel electrophoresis (SDS-PAGE) of purified secreted proteins (10 μg) in the culture filtrate. Arrows indicate ToxA (13.2 kDa).
(PNG)

S1 Table Table of PtrHp1, Tp and IR copies in Pleosporale genomes. (XLSX)

S2 Table M4 PtrHp1 element nucleotide (bp) overlap with M4 mRNA. (XLSX)

## References

[1] Moffat C, Santana MF. Diseases affecting wheat: tan spot In: O R, editor. Integrated disease management of wheat and barley: Burleigh dodds; 2018.

[2] Adhikari TB, Bai J, Meinhardt SW, Gurung S, Myrfield M, Patel J, et al Tsn1-mediated host responses to ToxA from Pyrenophora tritici-repentis. Mol Plant Microbe Interact. 2009;22(9):1056–68. 10.1094/MPMI-22-9-1056 . 19656041

[3] Tan KC, Oliver RP, Solomon PS, Moffat CS. Proteinaceous necrotrophic effectors in fungal virulence. Funct Plant Biol. 2010;37(10):907–12. 10.1071/Fp10067

[4] Tomas A, Feng GH, Reeck GR, Bockus WW, Leach JE. Purification of a Cultivar-Specific Toxin from Pyrenophora-Tritici-Repentis, Causal Agent of Tan Spot of Wheat. Mol Plant Microbe In. 1990;3(4):221–4. 10.1094/Mpmi-3-221

[5] Tuori RP, Wolpert TJ, Ciuffetti LM. Purification and immunological characterization of toxic components from cultures of Pyrenophora tritici-repentis. Mol Plant Microbe Interact. 1995;8(1):41–8. . 7772802 10.1094/mpmi-8-0041

[6] Ciuffetti LM, Tuori RP, Gaventa JM. A single gene encodes a selective toxin causal to the development of tan spot of wheat. Plant Cell. 1997;9(2):135–44. 10.1105/tpc.9.2.135 . 9061946 PMC156906

[7] Friesen TL, Ali S, Klein KK, Rasmussen JB. Population Genetic Analysis of a Global Collection of Pyrenophora tritici-repentis, Causal Agent of Tan Spot of Wheat. Phytopathology. 2005;95(10):1144–50. 10.1094/PHYTO-95-1144 . 18943466

[8] Manning VA, Chu AL, Steeves JE, Wolpert TJ, Ciuffetti LM. A host-selective toxin of Pyrenophora tritici-repentis, Ptr ToxA, induces photosystem changes and reactive oxygen species accumulation in sensitive wheat. Mol Plant Microbe Interact. 2009;22(6):665–76. 10.1094/MPMI-22-6-0665 . 19445591

[9] See PT, Marathamuthu KA, Iagallo EM, Oliver RP, Moffat CS. Evaluating the importance of the tan spot ToxA-Tsn1 interaction in Australian wheat varieties. Plant Pathol. 2018;67(5):1066–75. 10.1111/ppa.12835

[10] Friesen TL, Stukenbrock EH, Liu Z, Meinhardt S, Ling H, Faris JD, et al Emergence of a new disease as a result of interspecific virulence gene transfer. Nat Genet. 2006;38(8):953–6. 10.1038/ng1839 . 16832356

[11] McDonald MC, Ahren D, Simpfendorfer S, Milgate A, Solomon PS. The discovery of the virulence gene ToxA in the wheat and barley pathogen Bipolaris sorokiniana. Mol Plant Pathol. 2017;19(2):432–9. 10.1111/mpp.12535 . 28093843 PMC6638140

[12] Tan KC, Ferguson-Hunt M, Rybak K, Waters OD, Stanley WA, Bond CS, et al Quantitative variation in effector activity of ToxA isoforms from Stagonospora nodorum and Pyrenophora tritici-repentis. Mol Plant Microbe Interact. 2012;25(4):515–22. 10.1094/MPMI-10-11-0273 . 22250581

[13] Stukenbrock EH, McDonald BA. Geographical variation and positive diversifying selection in the host-specific toxin SnToxA. Mol Plant Pathol. 2007;8(3):321–32. 10.1111/j.1364-3703.2007.00396.x . 20507502

[14] Moolhuijzen P, See PT, Hane JK, Shi G, Liu Z, Oliver RP, et al Comparative genomics of the wheat fungal pathogen Pyrenophora tritici-repentis reveals chromosomal variations and genome plasticity. BMC Genomics. 2018;19(1):279 10.1186/s12864-018-4680-3 . 29685100 PMC5913888

[15] Moffat CS, See PT, Oliver RP. Generation of a ToxA knockout strain of the wheat tan spot pathogen Pyrenophora tritici-repentis. Mol Plant Pathol. 2014;15(9):918–26. 10.1111/mpp.12154 . 24831982 PMC6638721

[16] Antoni EA, Rybak K, Tucker MP, Hane JK, Solomon PS, Drenth A, et al Ubiquity of ToxA and absence of ToxB in Australian populations of Pyrenophora tritici-repentis. Australas Plant Path. 2010;39(1):63–8. 10.1071/Ap09056

[17] Sperschneider J, Gardiner DM, Dodds PN, Tini F, Covarelli L, Singh KB, et al EffectorP: predicting fungal effector proteins from secretomes using machine learning. New Phytol. 2016;210(2):743–61. 10.1111/nph.13794 . 26680733

[18] Petersen TN, Brunak S, von Heijne G, Nielsen H. SignalP 4.0: discriminating signal peptides from transmembrane regions. Nature Methods. 2011;8(10):785–6. Epub 2011/10/01. 10.1038/nmeth.1701 . 21959131

[19] Meile L, Croll D, Brunner PC, Plissonneau C, Hartmann FE, McDonald BA, et al A fungal avirulence factor encoded in a highly plastic genomic region triggers partial resistance to septoria tritici blotch. New Phytol. 2018 10.1111/nph.15180 . 29693722 PMC6055703

[20] Oliver R. Genomic tillage and the harvest of fungal phytopathogens. New Phytol. 2012;196(4):1015–23. 10.1111/j.1469-8137.2012.04330.x . 22998436

[21] Mair W, Lopez-Ruiz F, Stammler G, Clark W, Burnett F, Hollomon D, et al Proposal for a unified nomenclature for target-site mutations associated with resistance to fungicides. Pest Manag Sci. 2016;72(8):1449–59. 10.1002/ps.4301 . 27148866 PMC5094580

[22] Ren GX, Guo XP, Sun YC. Regulatory 3' Untranslated Regions of Bacterial mRNAs. Front Microbiol. 2017;8:1276 10.3389/fmicb.2017.01276 . 28740488 PMC5502269

[23] Hesketh J. 3′ UTRs and Regulation. eLS: John Wiley & Sons, Ltd; 2001.

[24] Chang SS, Zhang Z, Liu Y. RNA interference pathways in fungi: mechanisms and functions. Annu Rev Microbiol. 2012;66:305–23. 10.1146/annurev-micro-092611-150138 . 22746336 PMC4617789

[25] Ellwood SR, Syme RA, Moffat CS, Oliver RP. Evolution of three Pyrenophora cereal pathogens: recent divergence, speciation and evolution of non-coding DNA. Fungal Genet Biol. 2012;49(10):825–9. 10.1016/j.fgb.2012.07.003 . 22850609

[26] Momeni H, Aboukhaddour R, Javan-Nikkhah M, Razavi M, Naghavi MR, Akhavan A, et al Race Identification of Pyrenophora Tritici-Repentis in Iran. J Plant Pathol. 2014;96(2):287–94.

[27] Maclean DE, Aboukhaddour R, Tran VA, Askarian H, Strelkov SE, Turkington TK, et al Race characterization of Pyrenophora tritici-repentis and sensitivity to propiconazole and pyraclostrobin fungicides. Canadian Journal of Plant Pathology. 2017;39(4):433–43.

[28] Moreno MV, Stenglein S, Perello AE. Distribution of races and Tox genes in Pyrenophora tritici-repentis isolates from wheat in Argentina. Trop Plant Pathol. 2015;40(2):141–6. 10.1007/s40858-015-0011-2

[29] Ortiz R, Trethowan R, Ferrara GO, Iwanaga M, Dodds JH, Crouch JH, et al High yield potential, shuttle breeding, genetic diversity, and a new international wheat improvement strategy. Euphytica. 2007;157(3):365–84. 10.1007/s10681-007-9375-9

[30] Moffat CS, See PT, Oliver RP. Leaf yellowing of the wheat cultivar Mace in the absence of yellow spot disease. Australas Plant Path. 2015;44(2):161–6. 10.1007/s13313-014-0335-2

[31] Andrews S. FastQC 2011 [2016]. http://www.bioinformatics.babraham.ac.uk/projects/fastqc/.

[32] Jiang H, Lei R, Ding SW, Zhu S. Skewer: a fast and accurate adapter trimmer for next-generation sequencing paired-end reads. BMC Bioinformatics. 2014;15:182 10.1186/1471-2105-15-182 . 24925680 PMC4074385

[33] Bolger AM, Lohse M, Usadel B. Trimmomatic: a flexible trimmer for Illumina sequence data. Bioinformatics. 2014;30(15):2114–20. 10.1093/bioinformatics/btu170 . 24695404 PMC4103590

[34] Bankevich A, Nurk S, Antipov D, Gurevich AA, Dvorkin M, Kulikov AS, et al SPAdes: a new genome assembly algorithm and its applications to single-cell sequencing. J Comput Biol. 2012;19(5):455–77. 10.1089/cmb.2012.0021 . 22506599 PMC3342519

[35] Olson SA. EMBOSS opens up sequence analysis. European Molecular Biology Open Software Suite. Brief Bioinform. 2002;3(1):87–91. . 12002227 10.1093/bib/3.1.87

[36] Kent WJ. BLAT—the BLAST-like alignment tool. Genome Res. 2002;12(4):656–64. Epub 2002/04/05. 10.1101/gr.229202 Article published online before March 2002 [doi] . 11932250 PMC187518

[37] Edgar RC. MUSCLE: a multiple sequence alignment method with reduced time and space complexity. BMC Bioinformatics. 2004;5:113 10.1186/1471-2105-5-113 . 15318951 PMC517706

[38] Edgar RC. MUSCLE: multiple sequence alignment with high accuracy and high throughput. Nucleic Acids Res. 2004;32(5):1792–7. 10.1093/nar/gkh340 . 15034147 PMC390337

[39] Waterhouse AM, Procter JB, Martin DM, Clamp M, Barton GJ. Jalview Version 2—a multiple sequence alignment editor and analysis workbench. Bioinformatics. 2009;25(9):1189–91. 10.1093/bioinformatics/btp033 . 19151095 PMC2672624

[40] Slater GS, Birney E. Automated generation of heuristics for biological sequence comparison. BMC Bioinformatics. 2005;6:31 10.1186/1471-2105-6-31 . 15713233 PMC553969

[41] Gremme G, Steinbiss S, Kurtz S. GenomeTools: a comprehensive software library for efficient processing of structured genome annotations. IEEE/ACM Trans Comput Biol Bioinform. 2013;10(3):645–56. 10.1109/TCBB.2013.68 . 24091398

[42] Steinbiss S, Gremme G, Scharfer C, Mader M, Kurtz S. AnnotationSketch: a genome annotation drawing library. Bioinformatics. 2009;25(4):533–4. 10.1093/bioinformatics/btn657 . 19106120

[43] Quevillon E, Silventoinen V, Pillai S, Harte N, Mulder N, Apweiler R, et al InterProScan: protein domains identifier. Nucleic Acids Res. 2005;33(Web Server issue):W116–20. 10.1093/nar/gki442 . 15980438 PMC1160203

[44] Reuter JS, Mathews DH. RNAstructure: software for RNA secondary structure prediction and analysis. BMC Bioinformatics. 2010;11:129 10.1186/1471-2105-11-129 . 20230624 PMC2984261

[45] Manning VA, Pandelova I, Dhillon B, Wilhelm LJ, Goodwin SB, Berlin AM, et al Comparative genomics of a plant-pathogenic fungus, Pyrenophora tritici-repentis, reveals transduplication and the impact of repeat elements on pathogenicity and population divergence. G3 (Bethesda). 2013;3(1):41–63. 10.1534/g3.112.004044 . 23316438 PMC3538342

[46] Ellwood SR, Liu Z, Syme RA, Lai Z, Hane JK, Keiper F, et al A first genome assembly of the barley fungal pathogen Pyrenophora teres f. teres. Genome Biol. 2010;11(11):R109 10.1186/gb-2010-11-11-r109 . 21067574 PMC3156948

[47] Syme RA, Hane JK, Friesen TL, Oliver RP. Resequencing and comparative genomics of Stagonospora nodorum: sectional gene absence and effector discovery. G3 (Bethesda). 2013;3(6):959–69. 10.1534/g3.112.004994 . 23589517 PMC3689807

[48] Richards JK, Wyatt NA, Liu Z, Faris JD, Friesen TL. Reference Quality Genome Assemblies of Three Parastagonospora nodorum Isolates Differing in Virulence on Wheat. G3 (Bethesda). 2017 10.1534/g3.117.300462 . 29233913 PMC5919747

[49] Quinlan AR. BEDTools: The Swiss-Army Tool for Genome Feature Analysis. Curr Protoc Bioinformatics. 2014;47:11.2.1–34. 10.1002/0471250953.bi1112s47 . 25199790 PMC4213956

[50] Quinlan AR, Hall IM. BEDTools: a flexible suite of utilities for comparing genomic features. Bioinformatics. 2010;26(6):841–2. 10.1093/bioinformatics/btq033 . 20110278 PMC2832824

[51] Finn RD, Coggill P, Eberhardt RY, Eddy SR, Mistry J, Mitchell AL, et al The Pfam protein families database: towards a more sustainable future. Nucleic Acids Research. 2016;44(D1):D279–D85. 10.1093/nar/gkv1344 26673716 PMC4702930

[52] Ashburner M, Ball CA, Blake JA, Botstein D, Butler H, Cherry JM, et al Gene Ontology: tool for the unification of biology. Nature Genetics. 2000;25(1):25–9. 10.1038/75556 10802651 PMC3037419

[53] Carbon S, Dietze H, Lewis SE, Mungall CJ, Munoz-Torres MC, Basu S, et al Expansion of the Gene Ontology knowledgebase and resources. Nucleic Acids Research. 2017;45(D1):D331–D8. 10.1093/nar/gkw1108 27899567 PMC5210579

[54] Smith PK, Krohn RI, Hermanson GT, Mallia AK, Gartner FH, Provenzano MD, et al Measurement of Protein Using Bicinchoninic Acid. Anal Biochem. 1985;150(1):76–85. 10.1016/0003-2697(85)90442-7 3843705

[55] Rybak K, See PT, Phan HT, Syme RA, Moffat CS, Oliver RP, et al A functionally conserved Zn2 Cys6 binuclear cluster transcription factor class regulates necrotrophic effector gene expression and host-specific virulence of two major Pleosporales fungal pathogens of wheat. Mol Plant Pathol. 2017;18(3):420–34. 10.1111/mpp.12511 . 27860150 PMC6638278

