# Supplementary material for: Genomic distribution of a novel Pyrenophora tritici-repentis ToxA insertion element
Source: PLoS One. 2018 Oct 31;13(10):e0206586. doi: 10.1371/journal.pone.0206586 (PMC6209302; doi:10.1371/journal.pone.0206586)
Supplement: S1 Fig — (A) Clone product amplification for M14d, (B) Sanger read base signals for clone sequence of M14d, (C) Sequence alignment of M4 ToxA gene region and M14d clone 1 and clone 2, the clone 1 palindromic sequence is shown as a grey bar. (PDF) [file pone.0206586.s001.pdf]

A

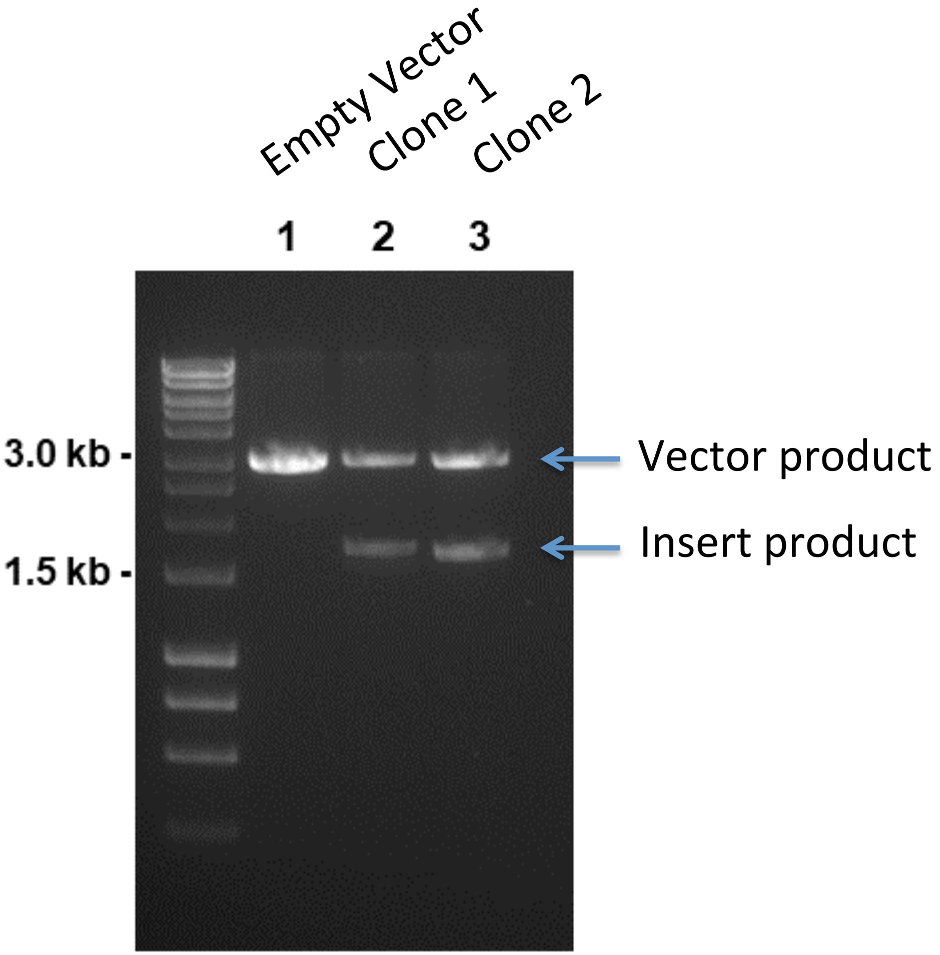

S1 Figure. Clonal product support.
A) Clone product amplification for M14d

B

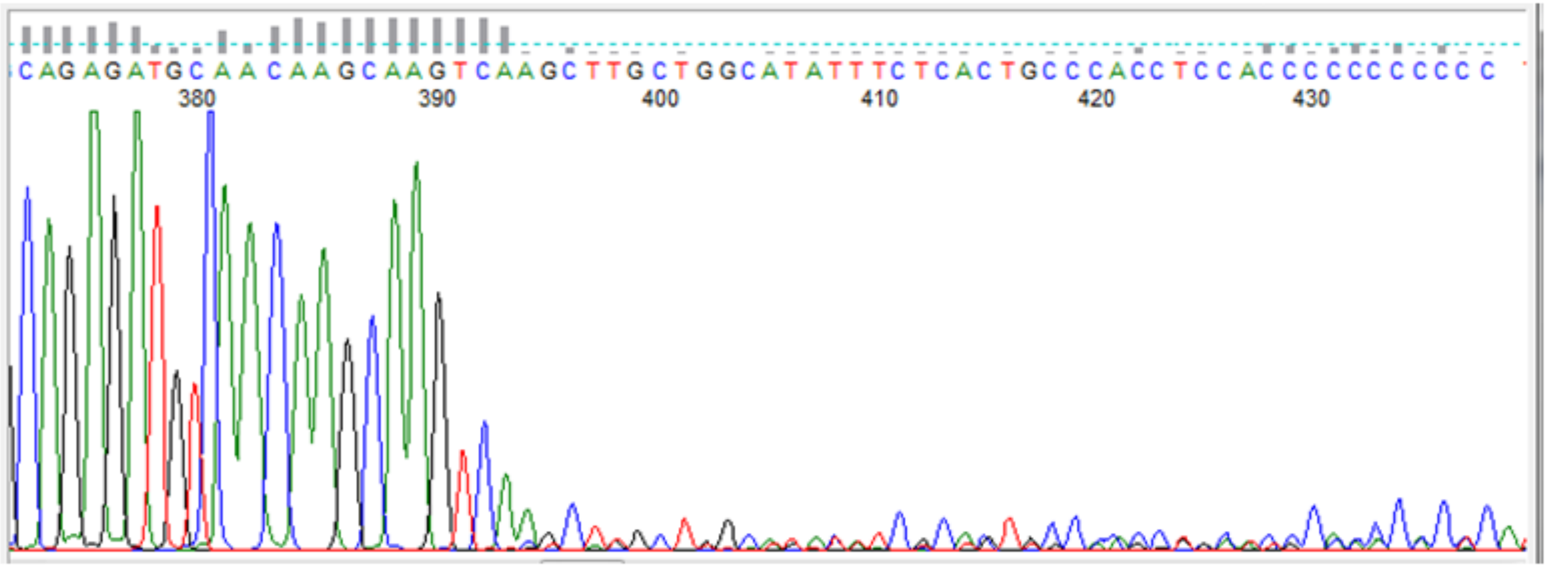

S1 Figure. Clonal product support.

B) Sanger read base signals for clone sequence of M14d

C

Palindromic region in Ptr ToxA locus

Clones containing larger amplicon derived from M14d New Zealand DNA

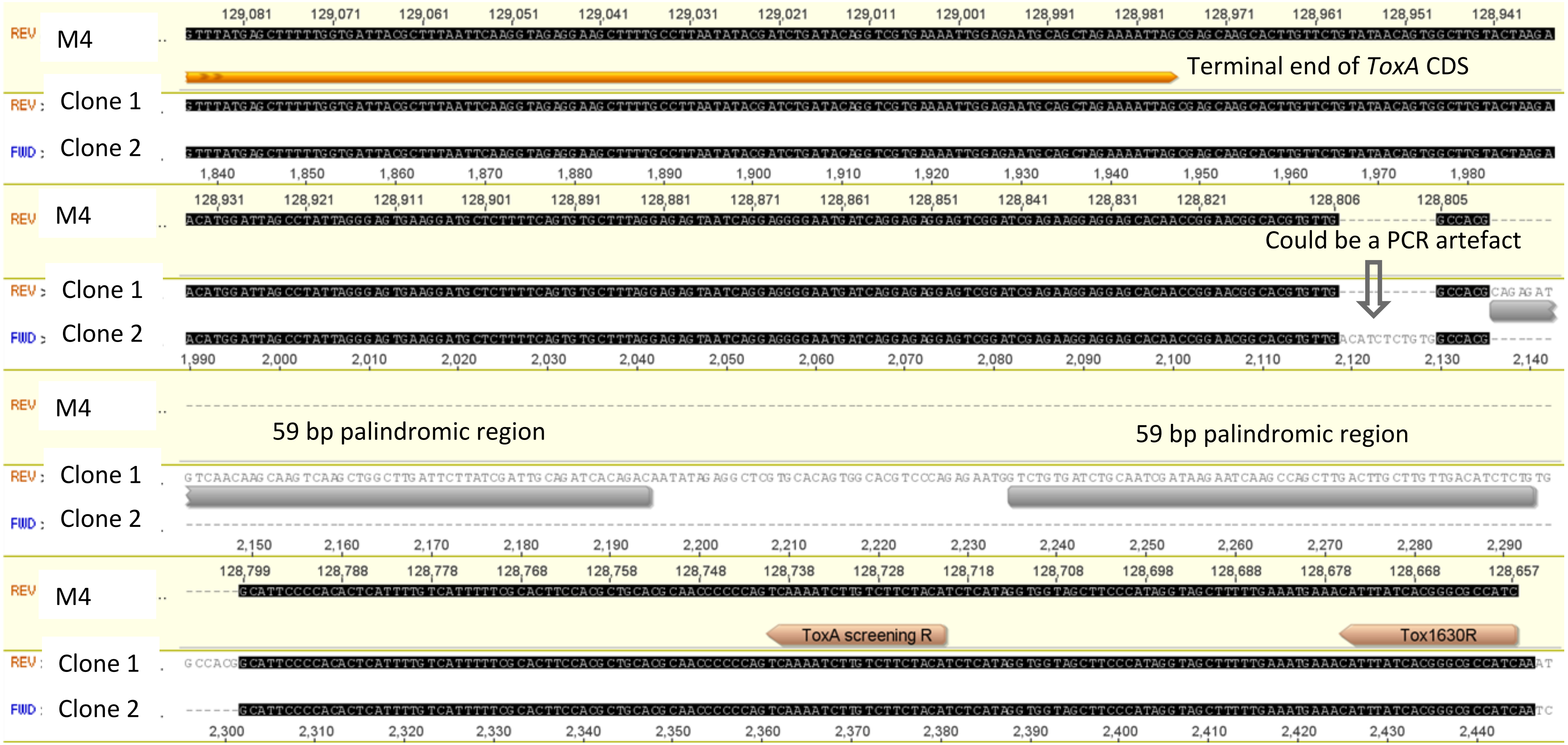

S1 Figure. Clonal product support

C) Sequence alignment of M4 *ToxA* gene region and M14d clone 1 and clone 2, the clone 1 palindromic sequence is shown as a grey bar.
