# Supplementary material for: Genomic distribution of a novel Pyrenophora tritici-repentis ToxA insertion element
Source: PLoS One. 2018 Oct 31;13(10):e0206586. doi: 10.1371/journal.pone.0206586 (PMC6209302; doi:10.1371/journal.pone.0206586)
Supplement: S2 Fig — A) Pyrenophora tritici-repentis (Ptr) ToxA region nucleotide multiple sequence alignment shows the 166bp sequence insertion in isolates EW4-4 and SN001C. B) Pyrenophora tritici-repentis (Ptr) ToxA region (~2kb) nucleotide sequence plot shows the 166bp sequence insertion in isolates CC142 ToxA 3’ UTR and downstream intergenic inverse repeat element (IR). a) CC142 self plot and b) CC142 on the horizontal axis and M4 on the vertical axis. CC142 ToxA mRNA UTRs (red) and CDS (green) are displayed on the bottom axis. (PDF) [file pone.0206586.s002.pdf]

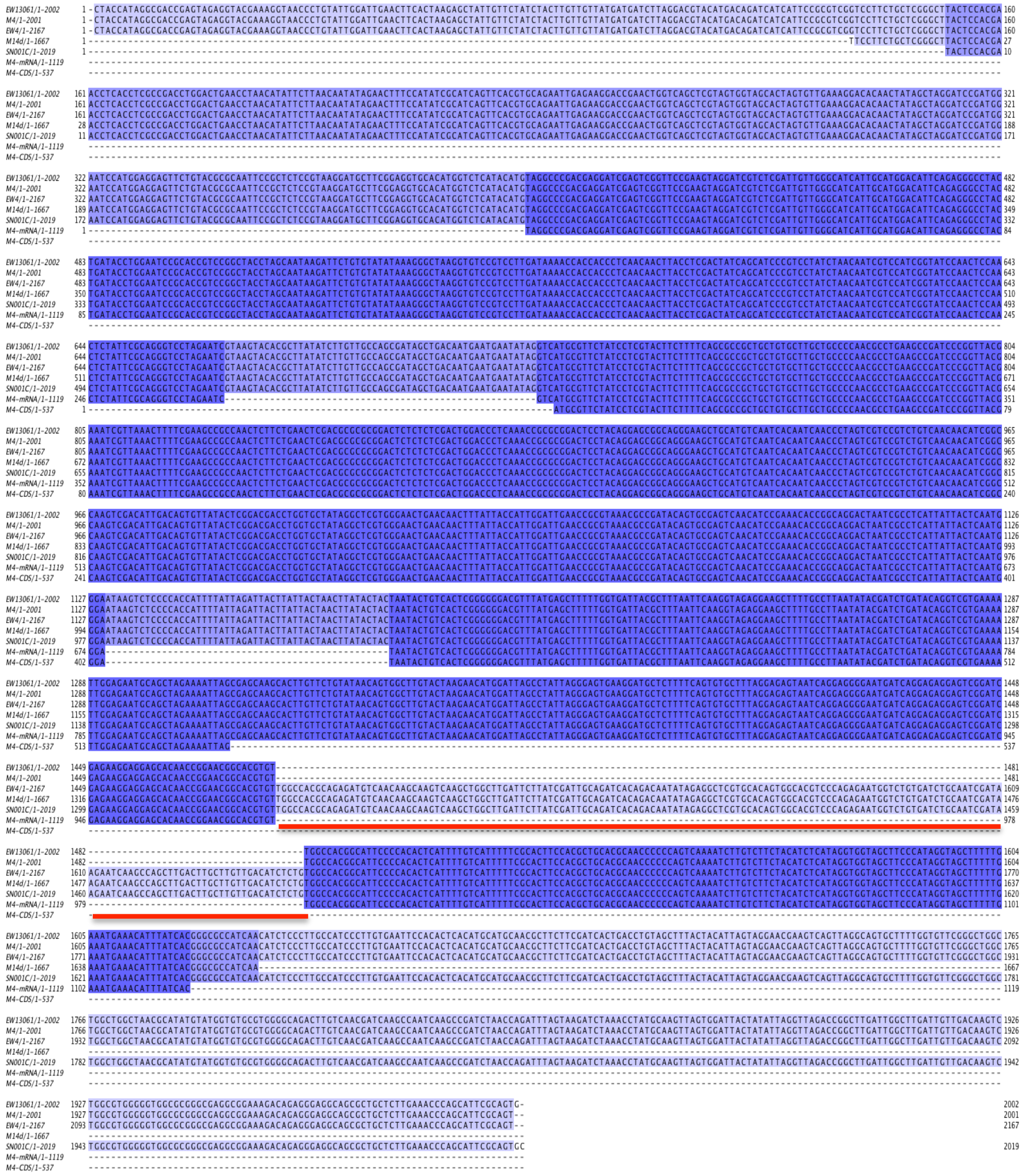

S2 A. *Pyrenophora tritici-repentis* (Ptr) *ToxA* region nucleotide multiple sequence alignment shows the 166bp sequence insertion in isolates EW4-4, M14d and SN001C

3'UTR insertion sequence

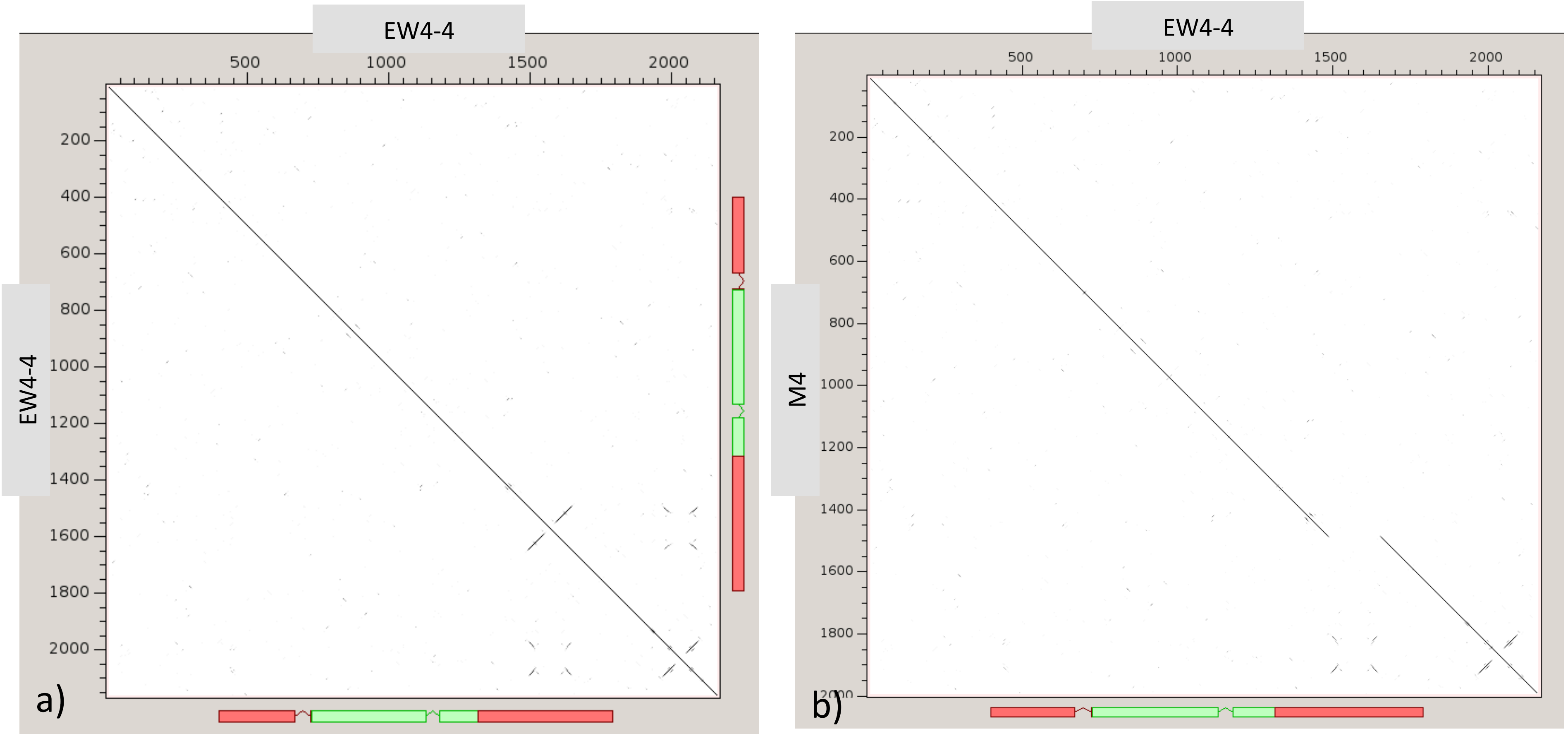

S2 B. *Pyrenophora tritici-repentis* (Ptr) *ToxA* region (~2kb) nucleotide sequence plot shows the 166bp sequence insertion in isolates EW4-4 *ToxA* 3' UTR and downstream intergenic inverse repeat element (IR). a) EW4-4 self plot and b) EW4-4 on the horizontal axis and M4 on the vertical axis. EW4-4 *ToxA* mRNA UTRs (red) and CDS (green) are displayed on the bottom axis
