# Supplementary figures and images for: Genomic distribution of a novel Pyrenophora tritici-repentis ToxA insertion element
Source: PLoS One. 2018 Oct 31;13(10):e0206586. doi: 10.1371/journal.pone.0206586 (PMC6209302; doi:10.1371/journal.pone.0206586)

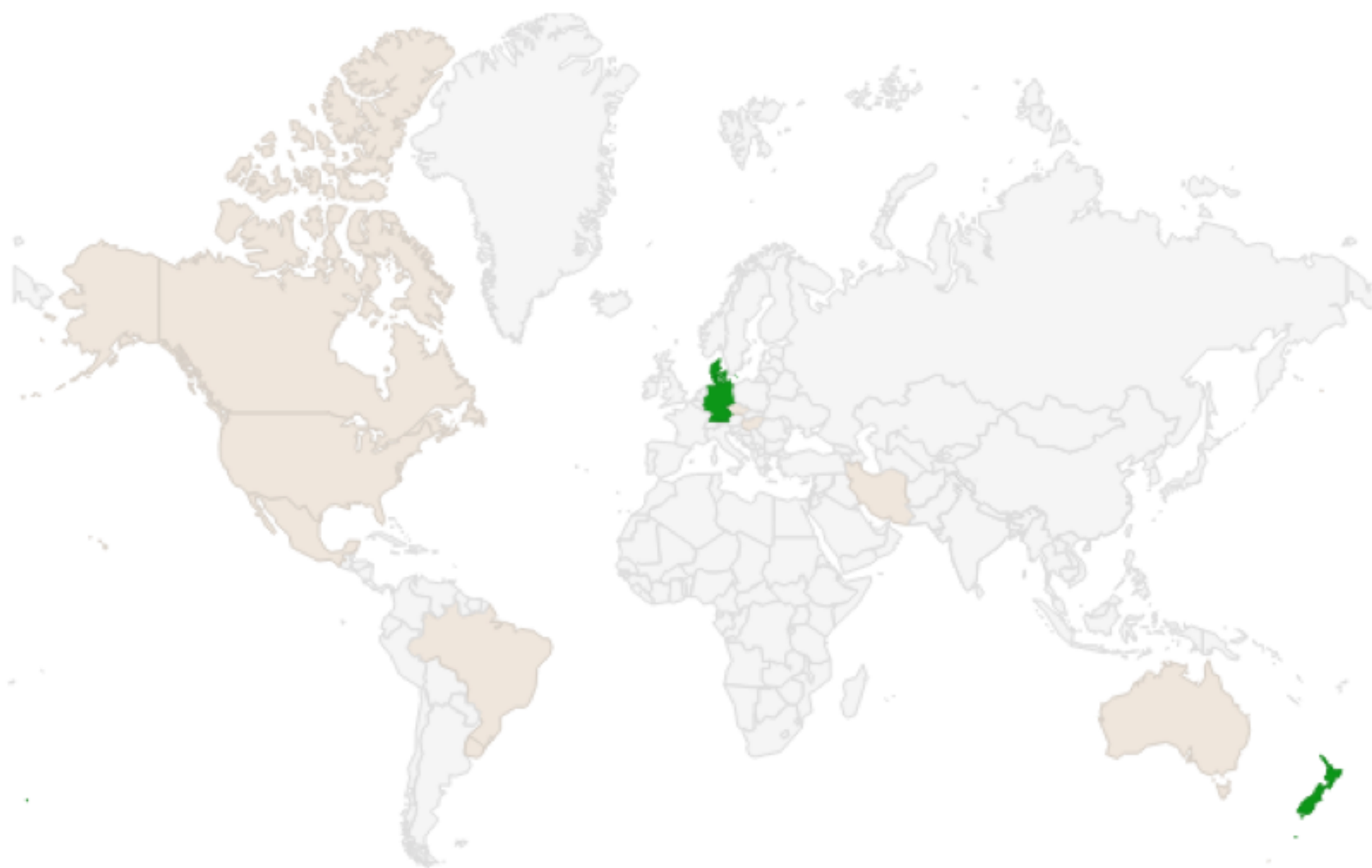

Supplement: S3 Fig — Countries with PtrHp1 detected in isolates are shown in red and in grey if not detected. (PDF) [file pone.0206586.s003.pdf]

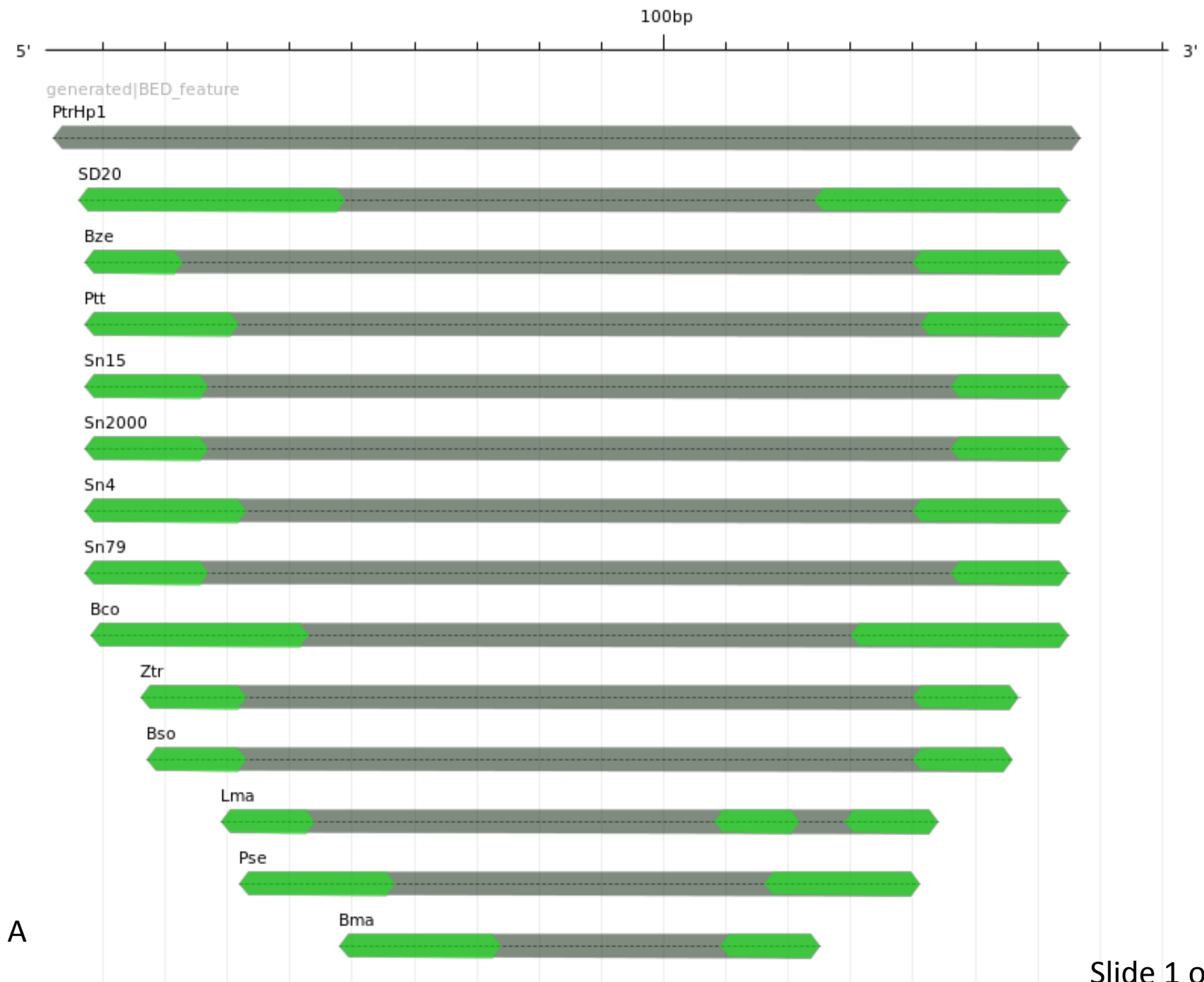

A

B

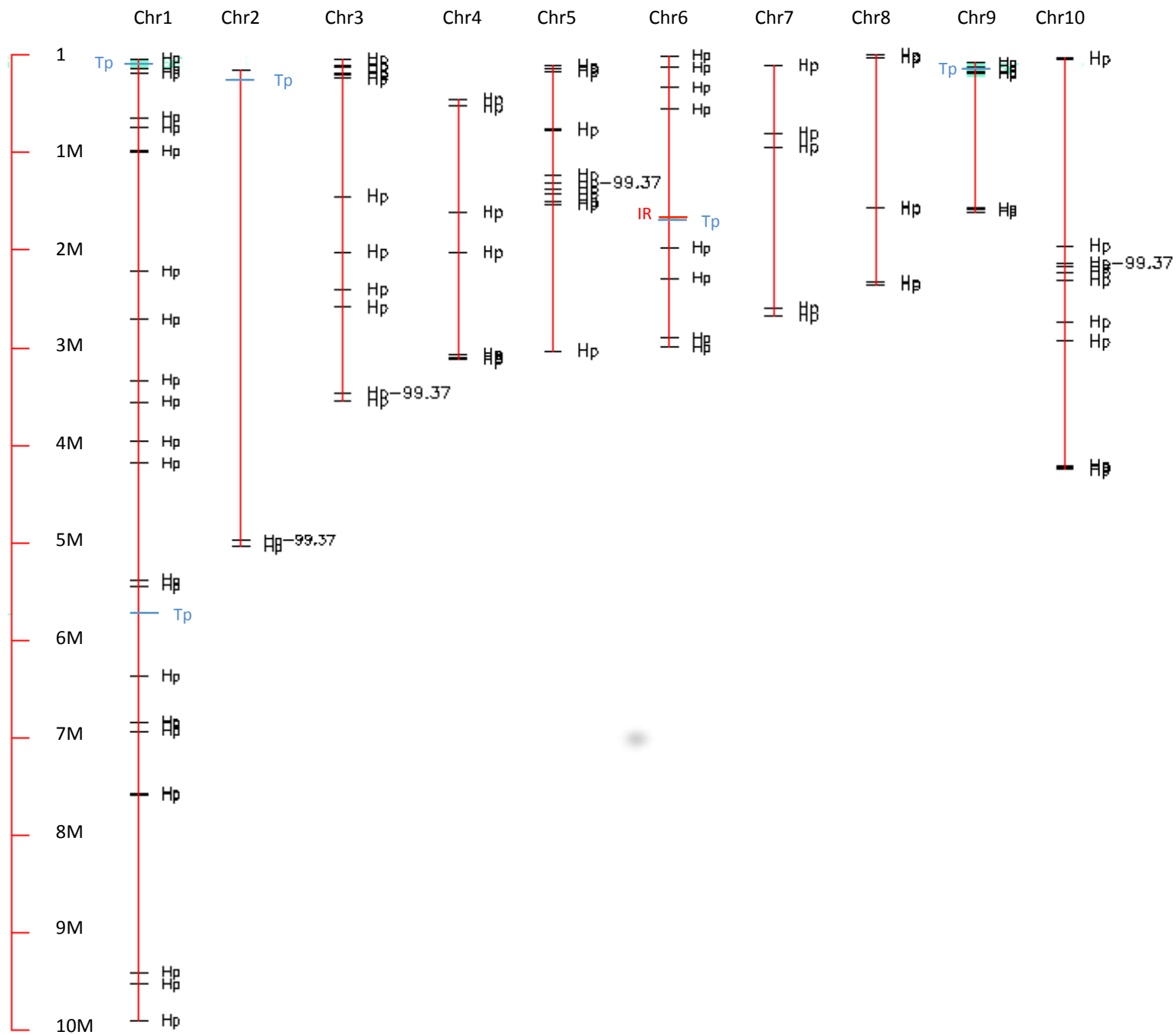

Supplement: S4 Fig — A) Pleosporales sequence similarity to PtrHp1. Ptr race 4 (SD20), B.zeicola (Bze), P. teres teres (Ptt), P. nodorum (Sn15, Sn2000, Sn4, Sn79), B. cookie (Bco), Z. tritici (Ztr), B. sorokiniana (Bso), L. maculans (Lma), P. seminiperda (Pse) and B. maydis (Bma). B) The distribution of Ptr elements PtrTp (Tp blue), the IR-IE (IR red) and PtrHp (Hp black) are shown in M4 genome. (PDF) [file pone.0206586.s004.pdf]

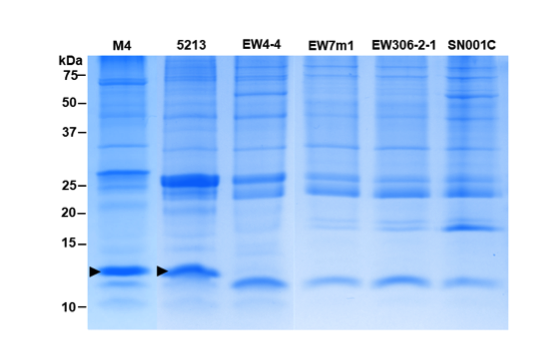

Supplement: S5 Fig — Arrows indicate ToxA (13.2 kDa). (PNG) [file pone.0206586.s005.png]
